# Gingival Fibroblasts Display Reduced Adhesion and Spreading on Extracellular Matrix: A Possible Basis for Scarless Tissue Repair?

**DOI:** 10.1371/journal.pone.0027097

**Published:** 2011-11-02

**Authors:** Fen Guo, David E. Carter, Anuradha Mukhopadhyay, Andrew Leask

**Affiliations:** 1 Department of Dentistry, University of Western Ontario, London, Ontario, Canada; 2 Robarts Research Institute, London, Ontario, Canada; 3 Department of Physiology and Pharmacology, University of Western Ontario, London, Ontario, Canada; Université de Technologie de Compiègne, France

## Abstract

Unlike skin, oral gingiva do not scar in response to injury. The basis of this difference is likely to be revealed by comparing the responses of dermal and gingival fibroblasts to fibrogenic stimuli. Previously, we showed that, compared to dermal fibroblasts, gingival fibroblasts are less responsive to the potent pro-fibrotic cytokine TGFβ, due to a reduced production of endothelin-1 (ET-1). In this report, we show that, compared to dermal fibroblasts, human gingival fibroblasts show reduced expression of pro-adhesive mRNAs and proteins including integrins α2 and α4 and focal adhesion kinase (FAK). Consistent with these observations, gingival fibroblasts are less able to adhere to and spread on both fibronectin and type I collagen. Moreover, the enhanced production of ET-1 mRNA and protein in dermal fibroblasts is reduced by the FAK/src inhibitor PP2. Given our previous observations suggesting that fibrotic fibroblasts display elevated adhesive properties, our data suggest that scarring potential may be based, at least in part, on differences in adhesive properties among fibroblasts resident in connective tissue. Controlling adhesive properties may be of benefit in controlling scarring in response to tissue injury.

## Introduction

In connective tissue, scars are areas of fibrosis that replace normal tissue after injury. Excessive scarring can obliterate normal tissue architecture, often resulting in organ failure and death. In fact, fibrotic disease is one of the largest groups of diseases for which there is no therapy. Although scarring is a natural response to wounding in skin, scarring is not observed in the oral cavity [1]. The cell type responsible for connective tissue repair and fibrosis is the fibroblast, the principal cells of stromal tissue [2]. Fibroblasts from different sites of the body display distinct and characteristic transcriptional patterns, suggesting that fibroblasts at different locations in the body can be considered distinct differentiated cell types [3]. Thus it can be postulated that differential responses of fibroblasts to fibrogenic stimuli underlie the basis of scarless tissue repair. Indeed, we have recently shown that gingival fibroblasts are less responsive to mechanical strain and the potent fibrogenic cytokine TGFβ due to a reduced production of endothelin-1 (ET-1) [4].

Previously, we have shown that fibroblasts isolated from fibrotic lesions of patients with the autoimmune connective tissue disease systemic sclerosis (SSc) show elevated adhesive signaling and an enhanced ability to adhere to and contract extracellular matrix [5]. Moreover, lesional SSc fibroblasts show elevated expression of components of focal adhesions, such as the integrins; a neutralizing anti-integrin β1 antibody alleviates the enhanced adhesive phenotype of this cell type [6]. Recently, it has been demonstrated that integrins are essential for the activation of latent TGFβ as well for the normal progression of tissue repair and fibrotic responses in vivo and in vitro [7]-[10]. Thus the elevated adhesive capacity of fibrotic (myo)fibroblasts embedded within scars appears to be an essential feature their fibrotic phenotype and blocking adhesive signaling could be a novel treatment for fibrosis.

Based on these observations, it is reasonable to hypothesize that a potential basis for the inability of gingiva to scar may be that the fibroblasts embedded within gingival show diminished adhesive properties compared to fibroblasts embedded within the dermis. In this report, we test this hypothesis by comparing both the gene expression profile and the adhesive properties of gingival and dermal fibroblasts.

## Methods

### Cell Culture

Gingival fibroblasts from three different human donors (HGF), identical to those previously described, were used for all experiments in this study [11]. Human dermal fibroblasts (HDF) were purchased (ATCC). Experiments were performed on cells between passage 5 and 7, as per previous publications using primary gingival fibroblasts [11]-[13]. Cells were cultured in DMEM, supplemented with 10% fetal bovine serum, 1% antibiotic-antimycotic (Invitrogen), 5% CO_2_ at 37^o^. Cells at 70% confluence were used for experiments.

### Real-time polymerase chain reaction

Real-time PCR was performed as previously described [4], [11], [14]. Total RNA was isolated (Trizol, Invitrogen) from cells at 70% confluence and then was reverse transcribed and amplified using TaqMan Assay-on-Demand primers (Applied Biosystems) and One-Step Master Mix (Applied Biosystems). Amplified sequences were detected using the ABI Prism 7900HT Sequence Detector (Perkin-Elmer-Cetus) according to the manufacturer's instructions. Triplicate samples were run. Expression values were standardized to values obtained with control 18S RNA primers, using the 2-^ΔΔCt^ method.

### ELISA

Secreted endothelin-1 (ET-1) levels were determined using cells at 70% confluence in triplicate using a Quantiglo Human Endothelin-1 Immunoassay (R&D Systems). 100 µl culture supernatants were used in the Quantiglo immunoassay, which was performed according to the manufacturer’s instructions. The standard curve is linear between 0.34 and 250 pg/ml of the ET-1 standard and was conducted for every assay.

### Western Blotting

Protein samples (100 µg/lane) from cells at 70% confluence were subjected to SDS-PAGE and transferred to PVDF membranes (Invitrogen). The resultant membranes were blocked with 5% milk-TBST for 1 hour at room temperature, and incubated with anti-FAK, anti-phospho-FAK, anti-p-JNK, anti-JNK, anti-p38, anti-p-p38, anti-JNK, anti-p-JNK (Cell Signaling), anti-α2- integrin, anti- α4- integrin and anti-β1-integrin antibody (Abcam) overnight at 4°C washed with TBST, incubated with appropriate secondary antibody (Jackson Immunoresearch, 1∶5000) conjugated to horseradish peroxidase, washed and visualized with ECL Western Blotting Detection Reagents (Amersham Biosciences). After stripping with Restore Western Blot Stripping Buffer (Pierce) for 20 minutes at room temperature, membranes were processed similarly with β-actin antibody (Sigma, 1∶10000 dilution) as a loading control.

### Expression Profiling

Expression profiling was conducted essentially as previously described [4] at the London Regional Genomics Centre (Robarts Research Institute, London, Ontario, Canada; http://www.lrgc.ca). RNA quality was assessed using the Agilent 2100 Bioanalyzer (Agilent Technologies Inc., Palo Alto, CA) and the RNA 6000 Nano kit (Caliper Life Sciences, Mountain View, CA). Single stranded complimentary DNA (sscDNA) was prepared from 200 ng of total RNA, end labeled and hybridized, for 16 hours at 45°C, to Human Gene 1.0 ST arrays. GeneChips were scanned with the GeneChip Scanner 3000 7G (Affymetrix, Santa Clara, CA) and probe level (.CEL file) data was generated using Affymetrix Command Console v1.1 and imported into Partek Genomics Suite (Partek, St. Louis, MO) using the RMA algorithm. The fold change between human dermal fibroblasts and gingival fibroblasts had to be at least 1.7 fold to identify a transcript as being altered. This list of genes was compiled and exported into DAVID (http://david.abcc.ncifcrf.gov/) for further analysis.

### Adhesion and spreading assays

Quantitative cell adhesion assays were performed essentially as previously described [5], [15], [16]. Wells of 96 well plates were incubated overnight, 4°C, with 1 µg/ml fibronectin or type I collagen (Sigma) in PBS. Wells were blocked for 1 hour in 1% BSA in PBS, room temperature. Fibroblasts at 80% confluence were harvested using 2 mM EDTA in PBS (20 min, room temperature), washed twice with DMEM serum-free medium containing 1% BSA (Sigma, St. Louis, MO) and resuspended in DMEM (2.5 × 10^4^ cells/100 µl). 2.5 × 10^4^ cells were placed into each well. At the times indicated, on-adherent cells were removed by washing with PBS. Adherent cells were quantified by incubation with 10 µl MTT (3-(4,5-dimethylthiazol-2-yl)-2,5-diphenyl tetrazolium bromide) solution for 4 h at 37°C, after which formazan reaction products in each well were dissolved in 100 µl of dimethyl sulphoxide and A_570_ was measured. Comparison of adhesive abilities was performed by using Student’s unpaired t-test. A p value<0.05 was considered as statistically significant.

Qualitative cell spreading assays were performed in parallel on cells cultured on glass coverslips. Cells were fixed in 4% paraformaldehyde, 0.5% Triton X-100, PBS for 20 minutes. Cells were then blocked in 5% normal goat serum, PBS (1 h room temperature), then incubated with anti-vinculin antibody (Sigma) overnight at 4°C, washed with PBST, incubated with Dylight 488-conjugated anti-mouse antibody (Jackson Immunoresearch) and rhodamine phalloidin (Cytoskeleton) and DAPI (1 µg/ml, 10 mins, room temperature, Molecular Probes). Cells were photographed using a Zeiss Axiophot and Northern Eclipse software (Empix). Stress fiber intensity and cell area was assessed Northern Eclipse software (Empix). Experiments were performed on three separate occasions.

### Statistical Analysis

Statistical tests were done using one-way ANOVA analysis of variance and Tukey’s *post hoc* test with GraphPad Software V.4 (Graphpad Software, La Jolla, CA, USA). P values less than 0.05 were taken to be significant.

## Results

### The expression of integrin α2 and α4 mRNA and protein are reduced in human gingival fibroblasts

**Figure 1:**
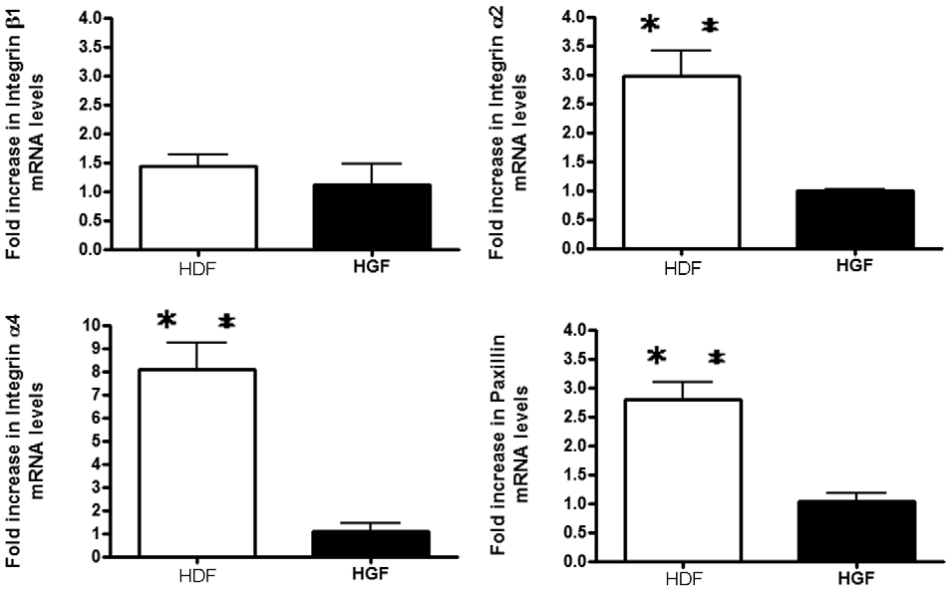
Expression of integrin α2, α4 and paxillin mRNAs are increased in human dermal fibroblasts (HDF) compared to human gingival fibroblasts (HGF). As described in Methods, equal numbers of fibroblasts were seeded into plates. Total RNA was harvested, and subjected to real-time PCR analysis with primers detecting the mRNAs indicated. Expression values are adjusted to those of controls (18S) run in parallel. Data shown are the average value from three independent experiments (3 replicates *per experiment*) ±SE. ** = p<0.01.

**Figure 2:**
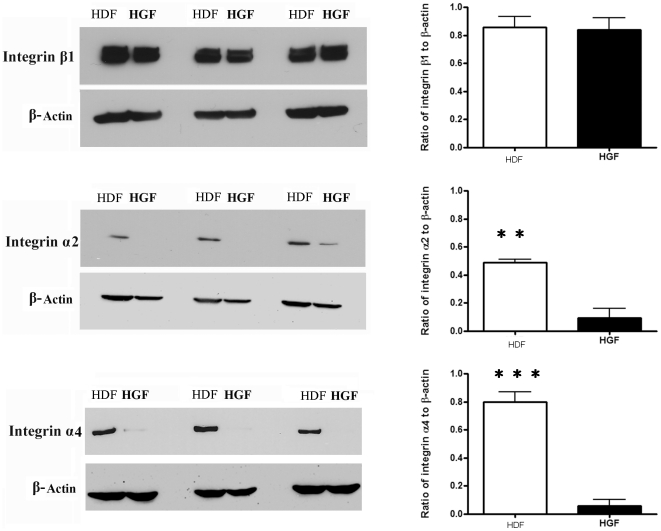
Expression of integrin α2 and α4 proteins are increased in human dermal fibroblasts (HDF) compared to human gingival fibroblasts (HGF). Cells were cultured until 80% confluence and incubated overnight in serum-free medium. Total protein was harvested, and subjected to Western blot analysis with the antibodies indicated. N = 3 average ±SE is shown * = p<0.05; ** = p<0.01.

We wished to identify inherent differences between dermal (HDF) and gingival (HGF) fibroblasts, thus we used genome-wide expression profiling to assess if differences in gene expression exist between the two cell types. Cells were cultured until 80% confluence. RNAs were then extracted, and subjected to Affymetrix gene profiling and cluster analysis. Using a 1.7 fold cutoff, approximately 800 genes were found to be over-expressed in HDF. Within these genes, there is a cluster of pro-adhesive mRNAs, including paxillin and integrins α2 and α4 (Table I). Real time PCR analysis verified our gene array data that these three mRNAs showed diminished expression in HGF compared to HDF (Fig 1). Intriguingly, expression of integrin β1 was not altered (Fig 1). Western blot analysis confirmed that integrins α2 and α4 showed reduced expression in HGF compared to HDF, whereas integrin β1 was not differentially expressed between HGF and HDF (Fig 2).

**Table 1:** Cluster analysis of mRNA (out of 831 total) upregulated in human dermal fibroblast (HDF) more than 1.7-fold than in human gingival fibroblasts (HGF). Average expression is shown.

Affymetrix ID	RefSeq	Gene name	Fold increase
*Cell adhesion*			
8175871	NM_000425	L1 cell adhesion molecule	12.5359
7962579	NM_001143668	adhesion molecule with Ig-like domain 2	1.7264
8022674	NM_001792	cadherin 2, type 1, N-cadherin	4.40822
8063796	NM_001794	cadherin 4, type 1, R-cadherin	1.7136
8019912	NM_032048	elastin microfibril interfacer 2	3.24879
8058765	NM_212482	fibronectin 1	1.7455
8044440	NM_153214	fibulin 7	1.75342
8105267	NM_002203	integrin, alpha 2	2.35478
8046695	NM_000885	integrin, alpha 4	2.20725
8046380	NM_000210	integrin, alpha 6	1.76366
8025601	NM_000201	intercellular adhesion molecule 1	2.2969
7952205	NM_006500	melanoma cell adhesion molecule	3.36377
8013341	NM_002404	microfibrillar-associated protein 4	2.87905
7925320	NM_002508	nidogen 1	2.68496
7961142	NM_002543	oxidized low density lipoprotein receptor 1	19.5212
7967021	BC052611	paxillin	1.81206
7971077	NM_006475	periostin, osteoblast specific factor	10.5474

(P<0.05).

### Human gingival fibroblasts show reduced FAK expression and FAK and p38 phosphorylation

**Figure 3:**
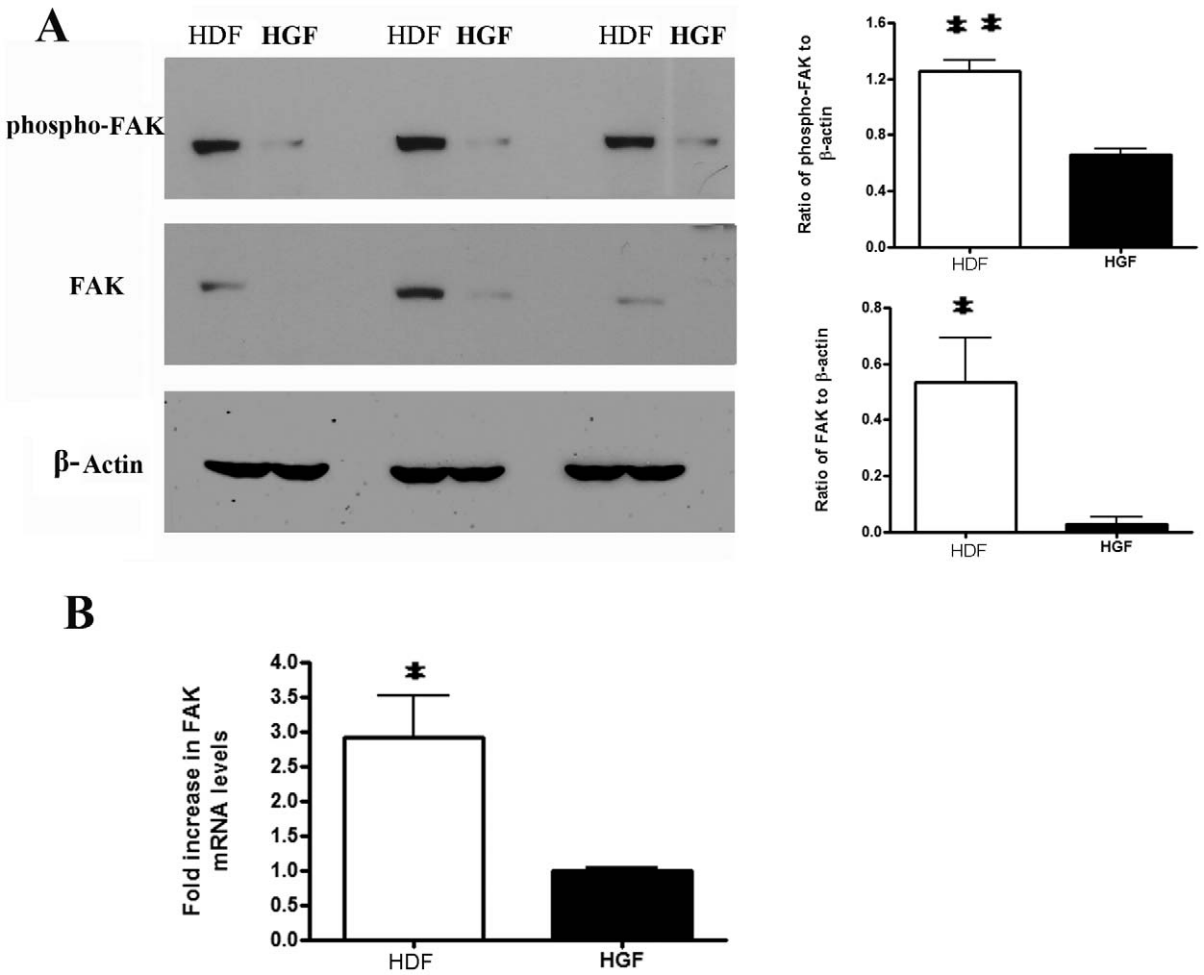
Focal adhesion kinase (FAK) expression and phosphorylation are increased in human dermal fibroblasts (HDF) compared to human gingival fibroblasts (HGF). (A) Cells were cultured until 80% confluence and incubated overnight in serum-free medium. Total protein was harvested, and subjected to Western blot analysis with the antibodies indicated. N = 3 average ±SE is shown * = p<0.05; ** = p<0.01. (B) Total RNA was harvested and subjected to real time PCR analysis with primers detecting FAK and 18S RNA. Data shown are the average value from three independent experiments (3 replicates *per experiment*) ±SE. * = p<0.05.

**Figure 4:**
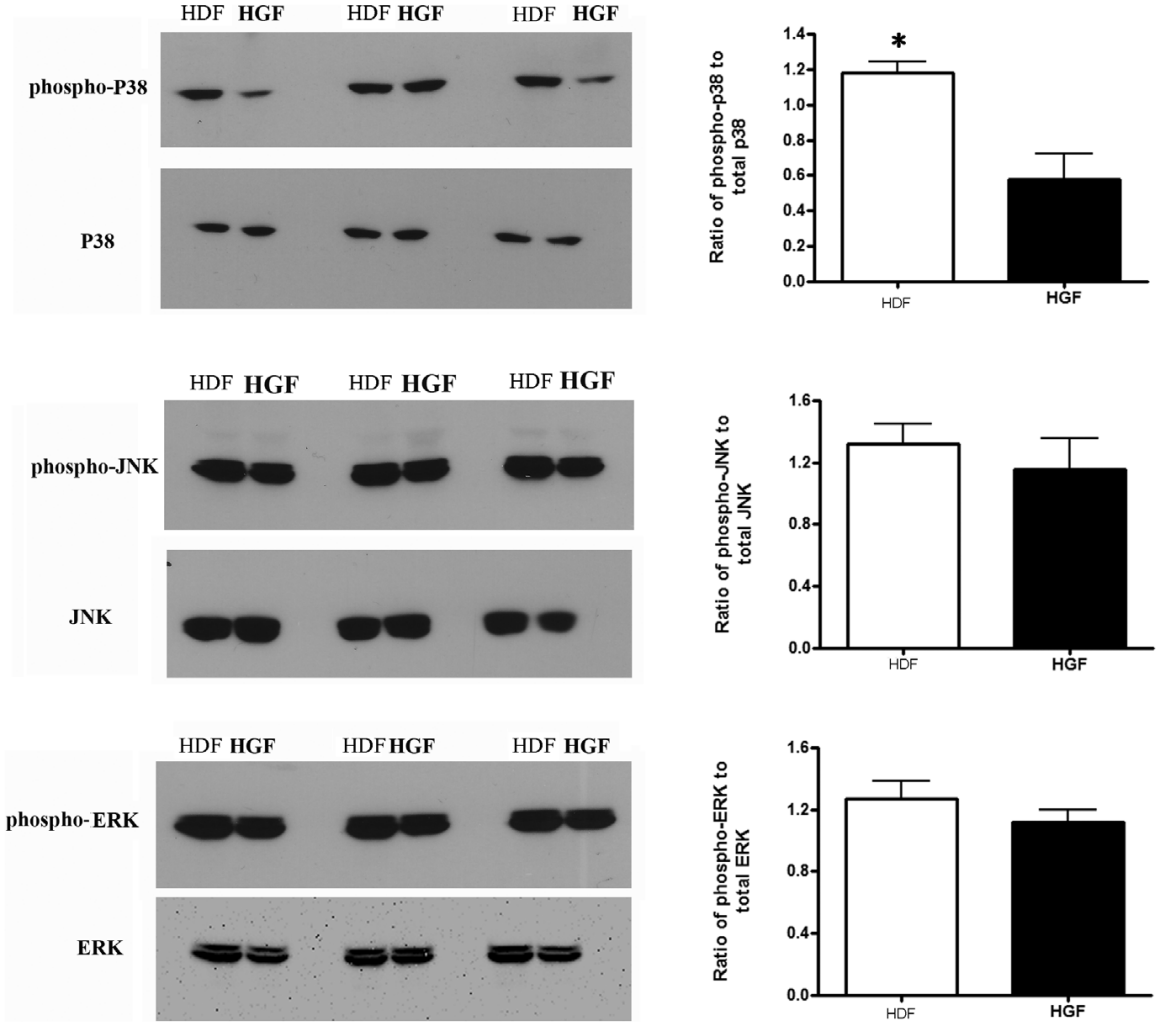
p38 phosphorylation is increased in human dermal fibroblasts (HDF) compared to human gingival fibroblasts (HGF). (A) Cells were cultured until 80% confluence and incubated overnight in serum-free medium. Total protein was harvested, and subjected to Western blot analysis with the antibodies indicated. N = 3 average ±SE is shown * = p<0.05; ** = p<0.01.

To assess whether HGF inherently show reduced activation of signaling pathways, we assessed whether phosphorylation of FAK was reduced in HGF compared to HDF. HGF and HDF were subjected to Western blot analyses with anti-phospho-FAK, anti-FAK and anti-β-actin antibodies. To our surprise, both phospho-FAK and total FAK protein were reduced in HGF (Fig 3A). Similarly, FAK mRNA expression was reduced in HGF (Fig 3B). Moreover, Western blot analyses revealed that HGF showed reduced p38 but not JNK or ERK phosphorylation (Fig 4). Collectively, these results indicate that reduced adhesive signaling may contribute to the phenotype of HGF.

**Figure 5:**
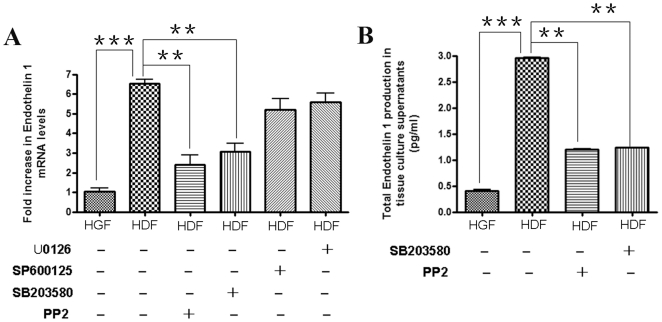
The elevated endothelin-1 (ET-1) production by human dermal fibroblasts is reduced by FAK/src and p38 inhibition. Cells were treated overnight with or without DMSO, PP2, U0126, SB203580, or the JNK inhibitor SP60012, as indicated (B) Conditioned media was subjected to ELISA to detect ET-1.** Data shown are the average value from three independent experiments (3 replicates** ***per experiment*** ) ±SE. *** = p<0.001; ** = p<0.01.

Compared to HGF, HDF show a significantly enhanced profibrotic response to TGFβ and strain and this is due to elevated ET-1 production by HDF [4]. To assess whether the elevated FAK phosphorylation in HDF contributed to the phenotype of these cells, we assessed whether the FAK/src inhibitor PP2 (10 µM) could reduce the overexpression of ET-1 in HDF. To perform this analysis, we first used real-time PCR to show that PP2 and the p38 inhibitor SB203580 (10 µM), but not the MEK/ERK inhibitor U0126 (10 µM) or the JNK inhibitor SP600125 (10 µM), significantly reduced ET-1 mRNA levels in HDF (Fig 5A). Similarly, a specific ELISA was used to show ET-1 protein (Fig 5B) expression in HDF was reduced in the presence of PP2 or SB. Collectively, these data suggest that the enhanced adhesive signaling observed in HDF, relative to HGF, contributes to the pro-fibrotic phenotype of this cell type.

### Human gingival fibroblasts show reduced adhesion and spreading on ECM

**Figure 6:**
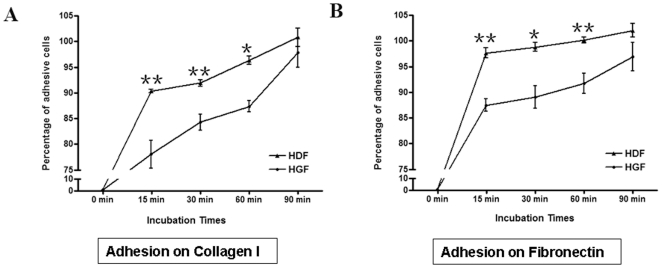
Human gingival fibroblasts are less adhesive than human dermal fibroblasts. As described in methods, equal numbers of human gingival fibroblasts (HGF) and human dermal fibroblasts (HDF) of equal passage were plated onto individual wells of 96 well plates that had been pre-coated with the ECM components fibronectin or type I collagen, as indicated. Unbound cells were removed by washing, an adherent cells at the times indicated were detected. Adhesion of HDF to ECM at 90 minutes was taken to represent 100% adhesion. **Data shown are the average value from 3 replicates** ***per experiment*** ) ±SE. * = p<0.05; ** = p<0.01.

**Figure 7:**
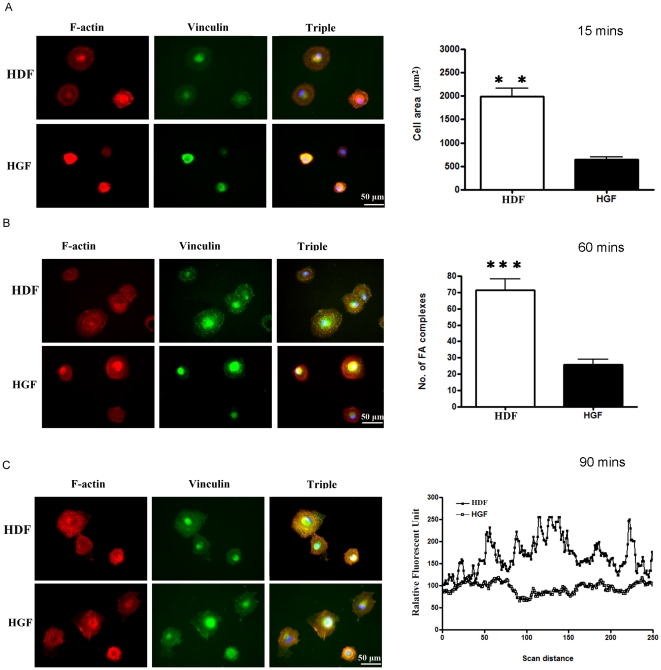
Human gingival fibroblasts spread less well on fibronectin than less human dermal fibroblasts. As described in methods, equal numbers of human gingival fibroblasts (HGF) and human dermal fibroblasts (HDF) of equal passage were plated onto glass coverslips that had been pre-coated with fibronectin. Unbound cells were removed by washing, an adherent cells at the times indicated were fixed and stained with rhodamine phalloidin (to detect actin, red), ant-vinculin antibody and appropriate FITC-conjugated secondary antibody (to detect focal adhesions, green) and DAPI (to detect nuclei, blue). Three separate experiments were conducted. (A) HDF spread better than HGF 15 minutes post-adhesion. Area of adherent cells was calculated using Northern Eclipse software. Data shown are the average value from 20 cells over three different experiments (total 60 cells) ±SE. ** = p<0.01. (B) HDF possess more focal adhesions (FA) than HGF 60 minutes post-adhesion. Number of focal adhesions per cell was calculated. Data shown are the average value from 20 cells over three different experiments (total 60 cells) ±SE. *** = p<0.001. (C) HDF have better defined actin stress fibers 90 minutes post-adhesion. The Northern Eclipse line tool was used to detect intensity of the red color (actin stress fibers). Representative traces across the cell are shown. Peaks represent location and intensity of stress fibers.

**Figure 8:**
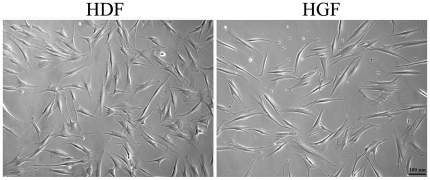
Human gingival fibroblasts possess a similar cell morphology to human dermal fibroblasts. Cells were cultured DMEM/10% FBS for 24 hours and live cells were subjected to light microscopy and photographed. A representative image is shown.

As, relative to HDF, HGF inherently showed reduced FAK expression and phosphorylation, we reasoned that the functional consequence of these alterations should be that HGF display reduced abilities to adhere and spread on ECM. Thus we compared the abilities of HDF and HGF to adhere to type I collagen and fibronectin in a standard adhesion assay. We found that, relative to HDF, HGF were less able to attach to type I collagen and fibronectin (Fig 6). Moreover, when cells were examined using microscopy and by staining with rhodamine-phalloidin (to detect actin) and anti-vinculin antibody (to detect focal adhesions), we found that, 15 minutes post-adhesion on fibronectin, HDF were more spread out than HGF (Fig 7A, surface area). By 60 minutes post-adhesion, HDF had more focal adhesions (Fig 7B, focal adhesions). Finally, by 90 minutes post-adhesion, HDF possessed more defined actin stress fibers (Fig 7C). Our cell spreading data paralleled and extended our cell attachment data and showed that, relative to HDF, HGF displayed elevated abilities to spread on ECM. Intriguingly, however, HGF and HDF examined 24 hours post-adhesion show a similar morphology, as revealed by light microscopy (Fig. 8). Collectively, these data indicate that HDF and HGF differ in their ability to attach to and spread on ECM.

## Discussion

Our manuscript is the first to show:

Gingival fibroblasts display reduced expression of integrins α2 and α4 and FAK; this difference is paralleled by reduced FAK and p-38 phosphorylation; the elevated ET-1 production observed in dermal fibroblasts is dependent on FAK/src and p-38 signaling; and gingival fibroblasts show significantly reduced ability to adhere to and spread on ECM.

Fibrosis, an often fatal condition characterized by excessive production and contraction of ECM in connective tissue, has no therapy [17], [18]. Unlike skin, gingivae do not scar [1]. As fibroblasts are the major cellular constituent of connective tissue, it is likely that differences in the phenotypes of gingival and dermal fibroblasts are likely to play a part in this distinction. Previously, we showed that, compared to fibroblasts isolated from clinically healthy tissue, fibrotic fibroblasts isolated from lesions of SSc patients show elevated expression of integrins, and enhanced adhesive signaling and ability [5]. In this report, we extend these studies and show that gingival fibroblasts possess reduced expression of integrins and focal adhesion kinase, and show reduced adhesive signaling and ability, relative to dermal fibroblasts. These results collectively indicate that differences in adhesive signaling potential of fibroblasts resident within connective tissue could be a major contributor to the extent of scarring observed clinically.

Compared to HDF, HGF inherently display a reduced expression of integrins and FAK. As might be expected, then, HDF show reduced endogenous activation of FAK (a kinase induced by adhesion, downstream of integrin engagement) [19] and p38 (a kinase activated downstream of integrin engagement) [20], [21]. A functional consequence of this difference is that, relative to HGF, HDF show impaired cell adhesion to ECM. Cell adhesion is multiphasic; there are two assays routinely used to assess this process [22]. One, the attachment assay, employs a colorimetric detection of bound cells [23]. The other, the spreading assay, employs microscopy to measure the flattening of adherent cells [24]. The first measures the actual number of attached cells, and reflects the inherent ability of cells to form connections to the ECM via integrins (i.e., it measures receptor-ligand binding). The second measures physical spreading (i.e., it measures changes in the fluxes through intracellular signaling pathways, and the resultant modulation of cytoskeletal assembly). In both types of adhesion assays, HGF show impaired adhesions; they show, relative to HDF, delayed attachment kinetics, reflecting reduced integrin expression by HGF, as well as delayed spreading kinetics, reflecting reduced adhesive signaling capacity of HGF, culminating in a diminished ability to quickly form focal adhesions and an actin cytoskeleton.

The α-SMA expressing myofibroblast is the effecter cell of normal tissue repair, scarring and fibrosis [2]. In normal fibroblasts, strain and TGFβ potently induce collagen type I and α-SMA expression [25]. However, neither strain nor TGFβ potently induce the expression of α-SMA or type I collagen mRNAs in gingival fibrobasts [4]. Previously, it has been shown that TGFβ and ET-1 synergize to promote myofibroblast differentiation of normal fibroblasts [26]. Surprisingly, we found that, compared to dermal fibroblasts, gingival fibroblasts do not express ET-1 [4]. Addition of ET-1 to gingival fibroblasts rescued the ability of this cell to response to TGFβ by inducing type I collagen and α-SMA [4]. Our results here, showing (a) that gingival fibroblasts have significantly lower levels of FAK activation and (b) that the overproduction of ET-1 by dermal fibroblasts is sensitive to the FAK/src inhibitor PP2 provide further mechanistic evidence that the elevated adhesive signaling observed in dermal fibroblasts, compared to gingival fibroblasts, contributes to the relative abilities of these two cell types to differentiate into myofibroblasts in response to TGFβ.

These data we believe to be important as they suggest that a reason that scarless tissue repair could occur resides in the reduced adhesive potential production of gingival fibroblasts embedded within connective tissue; modulating adhesive signaling might be a viable approach to controlling scarring in response to injury in the skin, and fibrotic disease.
